# Retrospective Evaluation of Pelvic and Acetabular Fracture Fixation Using the Stryker PRO Pelvis Next Generation Plating System

**DOI:** 10.7759/cureus.84334

**Published:** 2025-05-18

**Authors:** Robin M Litten, Doriann M Alcaide, Ryan N McIlwain, Clay A Spitler, Joseph P Johnson

**Affiliations:** 1 Department of Orthopedic Surgery, University of Alabama at Birmingham, Birmingham, USA

**Keywords:** fracture of acetabulum, orthopaedic trauma, pelvis fracture, plating system, surgical fixation

## Abstract

Introduction

Acetabular and pelvic fractures account for roughly 3% of all skeletal injuries and typically result from high-energy trauma. Surgical fixation is often required to restore pelvic stability and joint congruency. These procedures are technically demanding due to complex pelvic anatomy and limited access. Reconstruction plates are commonly used for their versatility in compression, tension, buttress, and antiglide functions.

The Stryker Pelvic PRO Next Generation plating system (Stryker, Portage, MI) offers implants for anterior and posterior fixation, designed to accommodate complex bony morphology. This study evaluates clinical and radiographic outcomes in patients treated with this implant system. We hypothesized that its use would yield rates of bone consolidation, nonunion, and adverse events comparable to the current literature.

Methods

With institutional review board approval, a retrospective review was conducted at a level I trauma center for patients treated with the Stryker PRO system between January and October 2024. Cases were identified using Current Procedural Terminology codes 27226, 27227, and 27228. Demographics, injury characteristics, surgical details, and outcomes were collected. Radiographic bone consolidation was defined as the presence of bridging callus in three out of four cortices with no visible fracture line or the disappearance of fracture lines, while clinical consolidation was defined as the patient achieving full weight-bearing status without pain. Delayed union was defined as the absence of radiographic healing by three months post-operatively, while nonunion was defined as persistent fracture at six months requiring surgical intervention or documented pain with visible fracture line. Descriptive statistics were performed using IBM SPSS Version 29.0.2.0.

Results

The study included 20 patients (mean age 44.6, 70% male) with pelvic or acetabular fractures. Most injuries resulted from motor vehicle accidents (70%), with common fracture types including AO 62.A1/62.B1 and posterior wall fractures. Radiographic and clinical consolidation were achieved in 80% of cases each, with 95% overall union. Seven adverse events occurred in five (25%) patients, including infection, heterotopic ossification, osteolysis, and avascular necrosis; 16.6% required reoperation. A total of 28 plates were used, most commonly spring plates (50%); 70% of patients required additional fixation.

Conclusion

The Stryker PRO implant system demonstrated rates of bone consolidation, nonunion, and adverse events comparable to the current literature.

## Introduction

Acetabular and pelvic fractures are complex injuries that occur in approximately 3% of all skeletal fractures, commonly resulting from high-energy trauma such as motor vehicle accidents or falls from height [1,2] Surgical fixation is often required and plays a crucial role in managing these fractures, with plating systems designed to provide stable fixation and allow for appropriate fracture healing [3,4]. These fractures present significant challenges due to the complex anatomy of the pelvis, the constraints of operating in a deep and narrow space, and the need for precise reduction to restore joint congruency and maintain stability [5-8].

In recent years, medical device companies have developed a wide range of implant systems to address the complex demands of pelvic and acetabular fracture fixation. As a result, there are now numerous plates and specialized implant options available to assist surgeons in achieving stable fixation [9]. The most commonly used plates are reconstruction plates [9]. They can be easily contoured and adapted to serve multiple roles, including compression, tension, buttress, and antiglide functions [9].

The Stryker Pelvic PRO Next Generation plating system (Stryker, Portage, MI) is one such option for fixation, offering plates designed for both anterior and posterior pelvic fixation. These implants allow for secure stabilization of fractures while accommodating the complex morphology of the pelvis and acetabulum, facilitating the goal of achieving anatomic reduction and optimal patient outcomes. The goal of this study was to describe radiographic and clinical outcomes in adults who underwent surgical fixation of the pelvis and/or acetabulum and received the Stryker Pelvic PRO Next Generation plating system. We hypothesize that the Stryker Pelvic PRO Next Generation plating system will achieve rates of bone consolidation, nonunion, and adverse events (AEs) comparable to those reported in the current literature for pelvic and acetabular fracture fixation.

## Materials and methods

**Figure 1:**
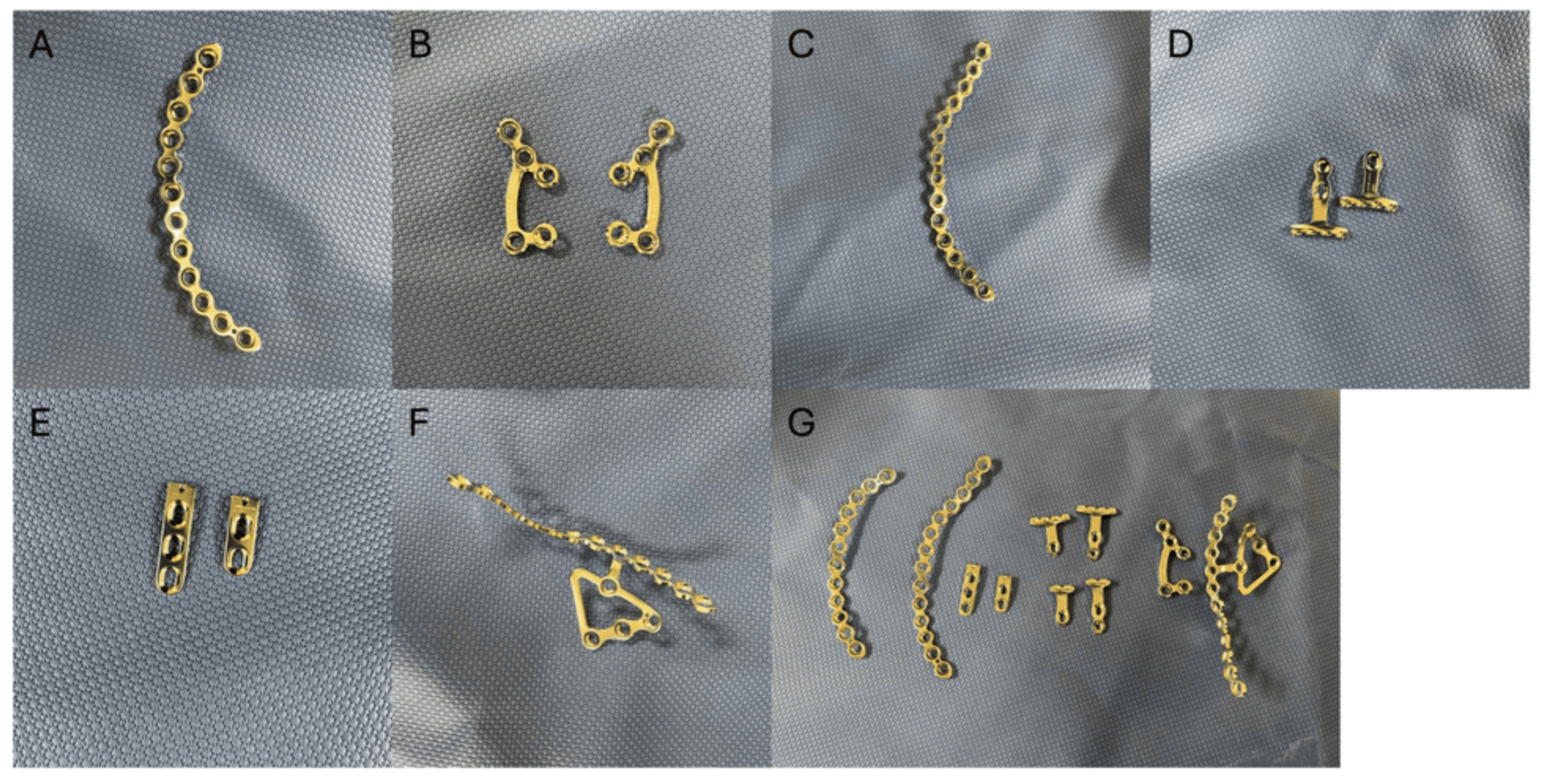
Components of the Pelvis PRO Next Generation plating system. (A) Pelvic brim plate. (B) Anterior sacroiliac plates. (C) Anterior column plate. (D) Posterior rim plate. (E) Spring plates. (F) Suprapectineal flex plate. (G) Complete assembly of the plating system.

After institutional review board approval, a retrospective review was performed at a single level I trauma center on all patients who underwent surgical fixation of pelvic or acetabular fractures between January 2024 and October 2024. Patients were identified through the institutional electronic medical record system using Current Procedural Terminology (CPT) codes 27226, 27227, and 27228. Patient records were manually reviewed to confirm eligibility for the study. Eligible patients received at least one Stryker PRO Pelvis Next Generation implant for fracture fixation (Figure 1).

Selection criteria included patients who were 18 years or older at the time of the index procedure, had previously received the PRO Next Generation plates in accordance with their indications for use (including fractures, non-unions, deformities, and malunions of the pelvic ring and acetabulum, or sacroiliac joint dislocations), and had sufficient follow-up to determine outcomes such as bone consolidation, nonunion, or implant failure, removal, or revision. Exclusion criteria were patients under 18 years of age at the time of the index procedure, patients with pelvis or acetabulum fractures who did not receive the PRO Next Generation implants, patients with inadequate follow-up to assess radiographic or clinical outcomes (i.e., consolidation status, presence of nonunion, implant failure, removal, or revision cannot be determined), patients with pathological pelvis or acetabulum fractures (e.g., metastatic disease or other bone pathology not related to trauma or congenital deformities), and patients with incomplete or missing operative records related to the index procedure.

Demographic and clinical data were collected, including age, sex, race, body mass index (BMI), comorbidities, mechanism of injury, fracture classification, surgical details (operative time, surgical approach, anesthesia time, and fluoroscopy time), length of hospital stay, and post-operative outcomes. The quality of the fracture’s reduction was determined using Matta’s radiographic criteria [10]. Preoperative, intraoperative, and follow-up visit documentation were reviewed to determine clinical and radiographic evidence of bone consolidation, implant failure, and/or complications. Radiographic bone consolidation was defined as the presence of bridging callus in three out of four cortices with no visible fracture line or the disappearance of fracture lines, while clinical consolidation was defined as the patient achieving full weight-bearing status without pain. Delayed union was defined as the absence of radiographic healing by three months post-operatively, while nonunion was defined as persistent fracture at six months requiring surgical intervention or documented pain with visible fracture line [11,12].

Patients with at least one follow-up visit sufficient to determine fracture healing status or implant failure/removal were included in the analysis. Descriptive statistics were generated to characterize the study population. Categorical variables were presented as numbers and percentages. Continuous variables were reported with means and standard deviations. Statistical analyses were performed on SPSS Version 29.0.2.0 (IBM Corp., Armonk, NY).

## Results

A total of 20 patients were included in this study, with a mean age of 44.6 years (20-78). The majority were male (70.0%), and the most common racial group was Caucasian (50.0%), followed by African American (40.0%). The mean BMI was 30.1 (20.7-48.3) kg/m². Tobacco use varied among patients, with 30.0% identified as current smokers, 25.0% identified as former smokers, and 45.0% reporting no history of smoking. Comorbid conditions included diabetes mellitus in 20.0% of patients and osteopenia in 15.0% (Table 1).

**Table 1:** Demographic and clinical characteristics of the study cohort BMI, body mass index; cm, centimeters; kg, kilograms; kg/m², kilograms per meter squared

Variable	n (%) or mean (SD)
Age (years)	44.55 (16.72)
Sex	Male: 14 (70.0%)
	Female: 6 (30.0%)
Race	Caucasian: 10 (50.0%)
	African American: 8 (40.0%)
	Other: 2 (10.0%)
Height (cm)	176.20 (7.90)
Weight (kg)	94.13 (29.03)
BMI (kg/m²)	30.12 (6.42)
Tobacco use	Current: 6 (30.0%)
	Former: 5 (25.0%)
	Never: 9 (45.0%)
Diabetes mellitus	Yes: 4 (20.0%)
	No: 16 (80.0%)
Osteopenia	Yes: 3 (15.0%)
	No: 17 (85.0%)

The most common mechanism of injury was a motor vehicle accident (70.0%), with motorcycle accidents, pedestrian injuries, and falls each contributing 10.0% of cases. Injury distribution was evenly split between right-sided and left-sided fractures (50.0% each). Regarding fracture classification, the most frequent AO fracture types were 62.A1 (posterior wall fracture of the acetabulum ± marginal impaction) (30.0%) and 62.B1 (transverse fracture of the acetabulum ± posterior wall fracture ± marginal impaction) (35.0%). The most common Letournel classification was transverse posterior wall fractures (50.0%), followed by posterior wall fractures (35.0%). The mean surgical time was 184.3 minutes (95-425 minutes), with an average anesthesia duration of 242.5 minutes (140-489 minutes). Intraoperative fluoroscopy time averaged 68.9 seconds (6-360 seconds). The mean length of hospital stay was 13.7 days (4-54 days) (Table 2).

**Table 2:** Injury patterns and operative details among PRO Next Generation Plate recipients SD, standard deviation

Variable	N (%) or mean (SD)
Mechanism of injury	Motor vehicle accident: 14 (70.0%)
	Motorcycle accident: 2 (10.0%)
	Pedestrian struck: 2 (10.0%)
	Fall: 2 (10.0%)
Side of injury	Right: 10 (50.0%)
	Left: 10 (50.0%)
AO classification	62.A1: 6 (30.0%)
	62.B1: 7 (35.0%)
	62.B2: 2 (10.0%)
	62.C1: 2 (10.0%)
	62.C3: 3 (15.0%)
Letournel classification	Posterior wall: 7 (35.0%)
	Transverse posterior wall: 10 (50.0%)
	T-type: 1 (5.0%)
	Both columns: 2 (10.0%)
	Anterior column: 2 (10.0%)
Surgical time (minutes)	184.25 (96.89)
Anesthesia time (minutes)	242.5 (96.15)
Fluoroscopy time (seconds)	68.9 (87.4)
Length of stay (days)	13.7 (14.1)

**Figure 2:**
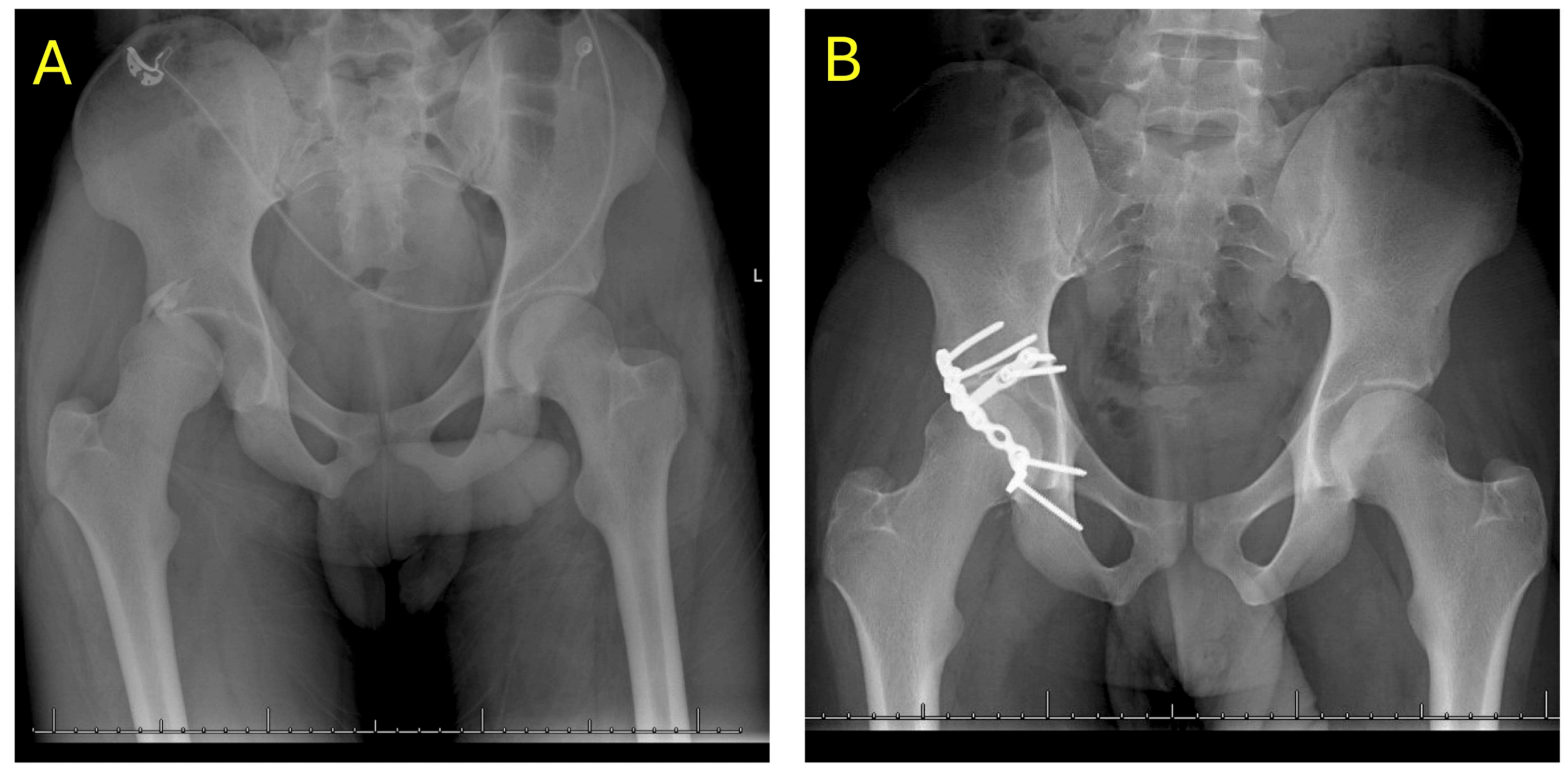
Serial radiographs of subject 5. (A) Injury film demonstrating initial fracture pattern. (B) Radiograph obtained 174 days post-operatively demonstrating interval healing and alignment.

**Figure 3:**
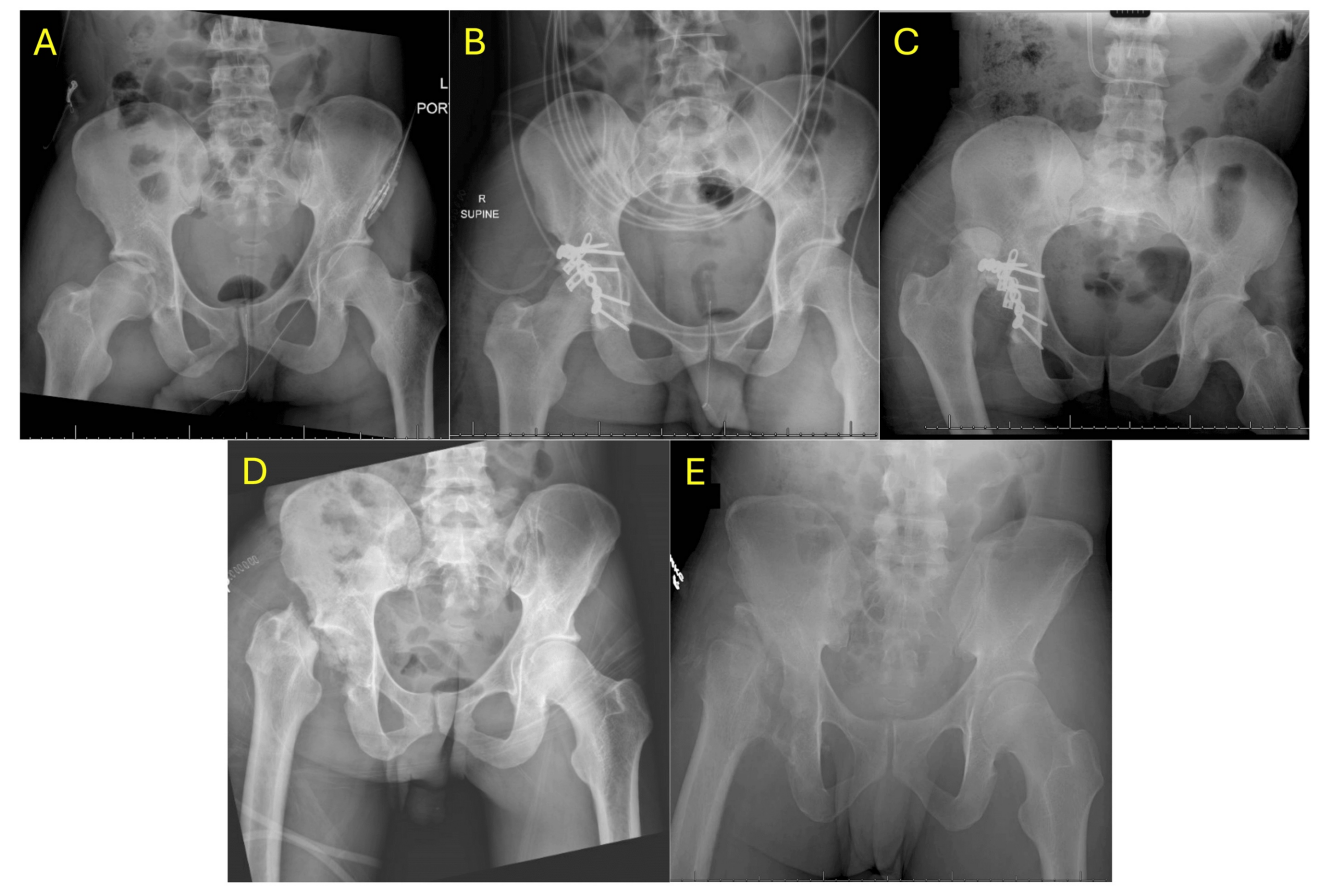
Serial radiographs of subject 1. (A) Injury film demonstrating initial fracture pattern. (B) Immediate post-operative radiograph following index fixation. (C) Radiograph obtained 26 days post-index surgery, prior to Girdlestone resection. (D) Immediate post-operative radiograph following the Girdlestone procedure. (E) Radiograph obtained 180 days post-index surgery demonstrating the post-operative course.

Radiographic and clinical consolidation were achieved in 80.0% of patients. As shown in Figure 2, subject 5 demonstrated clear evidence of radiographic consolidation, consistent with the trend observed in this cohort. The mean time to radiographic consolidation was 193 days (83-317 days), while clinical consolidation was achieved at an average of 108.00 days (51-275 days). Overall, 19 (95.0%) out of 20 patients achieved bone union, while one (5.0%) patient experienced nonunion (subject 1; shown in Figure 3). A total of 25.0% (5/20) of patients experienced at least one AE, and there were seven AEs reported among the 20 patients (Table 3).

**Table 3:** Bone healing outcomes and post-operative adverse events AE, adverse event

Variable	N (%) or mean (SD)
Follow-up (days)	187.9 (67.4)
Consolidation	19 (95.0%)
Radiographic consolidation	16 (80.0%)
Clinical consolidation	16 (80.0%)
Days after surgery: radiographic consolidation	193.00 (59.45)
Days after surgery: clinical consolidation	108.00 (49.90)
Patients with one or more AE	5 (25.0)
Time to AE (days)	85 (67.0)

Details regarding fracture type, surgical approach, and quality of reduction are presented by subject in Table 4. The most common surgical approach was the standard Kocher-Langenbeck, employed in nine (45.0%) cases. Other approaches included the Kocher approach (three cases, 15.0%), Stoppa approach (two cases, 10.0%), and various combinations or specific techniques such as the standard modified Stoppa with the standard Kocher-Langenbeck approach (one case, 5.0%), standard lateral window with anterior intrapelvic approach (one case, 5.0%), lateral window combined with the Pfannenstiel approach (one case, 5.0%), standard posterior Kocher approach (one case, 5.0%), posterior Kocher-Langenbeck approach (one case, 5.0%), and Pfannenstiel approach alone (one case, 5.0%). Regarding the quality of reduction, anatomic reductions were achieved in 10 (50.0%) cases, imperfect reductions were seen in eight (40.0%) cases, and poor reductions occurred in two (10.0%) cases (Table 4).

**Table 4:** Fracture types, surgical approaches, and quality of reduction in acetabular and pelvic fixation cases *Evaluated in accordance with Matta’s radiographic criteria [10]. LC2, lateral compression type II

Subject	Fracture type	Surgical approach	Quality of reduction*
1	Posterior wall acetabulum	Standard Kocher-Langenbeck	Poor
2	Both-column acetabulum	Standard modified Stoppa	Imperfect
		Standard Kocher-Langenbeck	
3	T-type acetabulum; LC2 pelvis	Stoppa	Anatomic
5	Posterior wall acetabulum	Standard Kocher-Langenbeck	Anatomic
6	Transverse posterior wall acetabulum	Standard Kocher-Langenbeck	Imperfect
7	Both-column acetabulum	Standard lateral window; anterior intrapelvic	Anatomic
8	Transverse posterior wall acetabulum	Standard Kocher-Langenbeck	Anatomic
9	Anterior column	Lateral window; Pfannenstiel	Imperfect
	Posterior hemitransverse acetabulum		
10	Posterior column	Standard Kocher-Langenbeck	Imperfect
	Posterior wall acetabulum		
11	Transverse posterior wall acetabulum	Standard Kocher-Langenbeck	Imperfect
12	Transverse posterior wall acetabulum	Standard posterior Kocher	Anatomic
14	Anterior column	Stoppa	Poor
	Posterior mid transverse acetabulum		
15	Posterior column	Kocher	Anatomic
	Posterior wall acetabulum		
16	Posterior wall acetabulum	Kocher	Anatomic
17	Transverse posterior wall acetabulum	Posterior Kocher-Langenbeck	Imperfect
18	Posterior wall acetabulum	Standard Kocher-Langenbeck	Anatomic
19	Posterior wall acetabulum	Standard Kocher-Langenbeck	Imperfect
20	Transverse posterior wall acetabulum	Pfannenstiel	Anatomic
21	Transverse posterior wall acetabulum	Standard Kocher-Langenbeck	Imperfect
22	Transverse posterior wall acetabulum	Kocher	Anatomic

The most common complications in this cohort were infection (10.0%), heterotopic ossification (HO) (15.0%), chondrolysis with post-traumatic arthritis (5.0%), and avascular necrosis (AVN) of the femoral head (5.0%). There was one occurrence of nerve damage (5.0%), though this was identified preoperatively and was not considered an AE related to this study. Among patients with AEs, one (20.0%) out of five required additional surgical intervention (Table 5).

**Table 5:** Subject-specific adverse events and outcomes AVN, avascular necrosis; I&D, irrigation and debridement; HO, heterotopic ossification; HWR, hardware removal; OA, osteoarthritis

Subject	Days post-operative	Adverse event	Reoperation
1	26	Infection with dislocation	Yes (I&D, HWR, Girdlestone)
1	35	HO	No
6	103	HO	No
9	209	Osteolysis, arthritis, fractures (post-traumatic OA)	No
15	45	Femoral head AVN	No
18	0 (intraoperative)	Infection	No
18	130	HO	No

Among the reported AEs, subject 1 developed a severe infection with dislocation 26 days postoperatively, necessitating a secondary operation consisting of irrigation and debridement, hardware removal, and a Girdlestone procedure, resulting in permanent disability. Notably, subject 1 exhibited evidence of HO on imaging performed 35 days post-operatively. Details of the post-operative course of subject 1 are summarized in Figure 3. Subject 6 developed HO 103 days post-operatively, which remains an ongoing issue but has not required revision surgery. Subject 9 developed chondrolysis associated with post-traumatic osteoarthritis 209 days post-operatively. This is still ongoing without necessitating revision surgery. Subject 15 was diagnosed with AVN of the femoral head 45 days post-operatively. This patient was subsequently scheduled for a total hip arthroplasty.

Subject 18 initially underwent fixation with reconstruction plates, but later experienced loss of fixation due to dislocation, requiring revision. During the revision procedure, the Pelvic PRO Next Generation plates were implanted, at which point an intraoperative infection was identified. The same subject (subject 18) subsequently developed HO 130 days post-operatively.

A total of 28 PRO Next Generation pelvic plates were used among the 20 patients, with each patient receiving an average of 1.4 PRO Next Generation pelvic plates. The most commonly used plate was the spring plate, which accounted for 50.0% (14/28) of all PRO Next Generation pelvic plates, followed by the posterior rim plate, 25.0% (7/28), and the suprapectineal flex plate, 21.4% (6/28). The anterior sacroiliac plate was the least frequently used, making up 3.6% (1/28) of all PRO Next Generation pelvic plates. On average, spring plates and posterior rim plates were secured with 1.4 screws each, while suprapectineal flex plates required 6.5 screws, and anterior sacroiliac plates were fixed with 4.0 screws per plate. Bending of plate was not required of any of the Pelvic PRO Next Generation plates. Additional fixation devices, including reconstruction plates and H-plates, were used in 70.0% (14/20) of patients. In all cases, the indication for the use of the plate was a fracture of the pelvis or acetabulum (Table 6).

**Table 6:** Plate configurations and supplementary implants by subject SI, sacroiliac

Subject	Stryker PRO plates	Supplemental plates
1	2 spring plates	8-hole reconstruction plate
2	Spring plate	11-hole reconstruction plate
	Suprapectineal flex plate	4-hole flex plate
3	Suprapectineal flex plate	-
5	Posterior rim plate	7-hole reconstruction plate
6	Spring plate	7-hole reconstruction plate
		8-hole reconstruction plate
7	Suprapectineal flex plate	8-hole reconstruction plate
		6-hole reconstruction plate
8	Posterior rim plate	H-plate
	Anterior SI plate	7-hole reconstruction plate
	Posterior rim plate	-
9	Suprapectineal flex plate	-
10	2 spring plates	6-hole reconstruction plate
		9-hole reconstruction plate
11	2 spring plates	8-hole reconstruction plate
		10-hole reconstruction plate
		H-plate
12	Posterior rim plate	5-hole reconstruction plate
	Spring plate	7-hole reconstruction plate
14	Suprapectineal flex plate	12-hole reconstruction plate
15	2 spring plates	7-hole reconstruction plate
		9-hole reconstruction plate
		4-hole reconstruction plate
16	Spring plate	-
17	Posterior rim plate	7-hole reconstruction plate
18	Spring plate	-
19	Posterior rim plate	-
20	Suprapectineal flex plate	-
21	Spring plate	8-hole reconstruction plate
		H-plate
22	Posterior rim plate	5-hole reconstruction plate
		6-hole reconstruction plate

## Discussion

In this series, the Stryker Pelvic PRO Next Generation plating system demonstrated fracture consolidation rates of 95.0% when used for the fixation of acetabular and pelvic fractures and an AE rate of 25.0%. The system was most commonly used for transverse posterior wall fractures (50.0%).

In the current study, 19/20 (95.0%) of patients achieved fracture union, and one (5.0%) patient (subject 1) failed to achieve fracture consolidation. Although malunion and nonunion following surgical treatment of acetabular and pelvic ring fractures are relatively rare, they pose significant management challenges when they occur [13-15]. The incidence of these complications is difficult to define precisely, in part due to their low frequency as well as inconsistent definitions across studies [13,14,16]. Mostert et al. reported an incidence of 3.4% (8/233) in individuals with pelvic fractures [14]. With acetabular fractures, the incidence ranges in the literature from 0% to 1.8% [10,16-18,20].

Subject 1 sustained a posterior wall acetabulum fracture and subsequently developed a post-operative infection complicated by hip dislocation. Intraoperatively, it was noted that there was no consolidation of the patient’s posterior wall fracture, and despite surgical intervention with irrigation and debridement, the patient ultimately required hardware removal and conversion to a Girdlestone procedure (Figure 3). In this cohort, this was the only report of nonunion using the Pelvic PRO Next Generation plating system. This case illustrates the profound impact that post-operative complications can have on fracture healing and emphasizes the importance of vigilant monitoring, particularly in high-risk patients [20,21].

The infection rate in this cohort was 10.0% (2/20 patients), which is slightly higher than the previously reported rates for pelvic and acetabular fracture fixation (5.0-7.0%) [22-26]. However, given the limited sample size, these findings may not be fully representative. The incidence of specific complications in this cohort, particularly HO in 15.0% of patients and femoral head AVN in 5.0%, falls within or below the range previously reported in the literature [27-31]. Giannoudis et al. reported HO rates of up to 25.0%, depending on the approach used and prophylactic measures taken, and AVN rates ranging from 5.0% to 10.0% [27]. The relatively low rates observed in this series may be reflective of advancements in surgical technique, perioperative care, or the stabilizing effect of the Stryker Pelvic PRO Next Generation plating system; however, the small sample size limits the generalizability of these findings and may also contribute to the observed complication rates.

Procaccini et al. have also explored this plating system, demonstrating the successful application of a three-dimensional anatomical preshaped suprapectineal plate, which, when positioned along the pelvic brim, provided the primary mechanical support for acetabular fracture fixation in their study cohort [32]. In their cohort of 33 patients, 91.0% reported high satisfaction with their functional outcomes during activities of daily living.

In our study, the assessment of reduction quality revealed that anatomic reductions were achieved in 10 (50.0%) cases, while imperfect reductions were seen in eight (40.0%) cases, and poor reductions occurred in two (10.0%) cases. To contrast, in their cohort of 80 patients, Kumar et al. observed 63.5% anatomic reductions, 22.2% imperfect reductions, and 14.4% poor reductions [33]. These differences in reduction quality could be influenced by the smaller sample size in our study (20) compared to Kumar et al.’s larger cohort of 80. Furthermore, the standard Kocher-Langenbeck approach was used in all cases in their study, compared to 45% of cases in this cohort.

This plating system is also useful for combining multiple plates to achieve ideal fixation, particularly in complex fracture patterns. In this series, seven patients required more than one Pelvic PRO Next Generation plate. The use of multiple plates was most common in posterior column fractures, posterior wall fractures, transverse posterior wall fractures, and both-column fractures, reflecting the need for enhanced stability in these injury patterns. The ability to use multiple plates within the same system allows for customized fixation strategies based on fracture morphology, which may contribute to improved stability and consolidation.

This study has limitations that should be considered when interpreting the results, including its retrospective design and small sample size of 20 patients, which may limit the generalizability and statistical power of the findings. Additionally, the relatively short follow-up period (187.9 days) may not capture long-term complications or outcomes. Future studies with larger, multicenter cohorts and longer follow-up are needed to better assess the long-term effectiveness and safety of the Stryker Pelvic PRO Next Generation plating system.

Overall, the findings suggest that in the absence of major complications, the Stryker Pelvic PRO Next Generation plating system was effective in achieving fracture consolidation, particularly in posterior wall fractures. Further studies with larger sample sizes and longer follow-up are needed to better assess long-term outcomes and complication rates associated with this fixation system.

## Conclusions

This retrospective study demonstrates that the Stryker Pelvic PRO Next Generation plating system is a viable option for the surgical management of pelvic and acetabular fractures, with comparable union rates to those previously reported and effective stabilization across a range of complex fracture patterns without requiring intraoperative plate bending. However, a notable proportion of patients experienced AEs, some of which necessitated reoperation and were potentially related to the device or procedure. Further prospective studies with larger cohorts are warranted to validate these findings, optimize patient selection, and refine strategies to mitigate complications.
